# Continuous, noninvasive wireless monitoring of flow of cerebrospinal fluid through shunts in patients with hydrocephalus

**DOI:** 10.1038/s41746-020-0239-1

**Published:** 2020-03-06

**Authors:** Siddharth R. Krishnan, Hany M. Arafa, Kyeongha Kwon, Yujun Deng, Chun-Ju Su, Jonathan T. Reeder, Juliet Freudman, Izabela Stankiewicz, Hsuan-Ming Chen, Robert Loza, Marcus Mims, Mitchell Mims, KunHyuck Lee, Zachary Abecassis, Aaron Banks, Diana Ostojich, Manish Patel, Heling Wang, Kaan Börekçi, Joshua Rosenow, Matthew Tate, Yonggang Huang, Tord Alden, Matthew B. Potts, Amit B. Ayer, John A. Rogers

**Affiliations:** 1 Department of Materials Science and Engineering, Frederick Seitz Materials Research Laboratory, University of Illinois at Urbana-Champaign, Urbana, IL 61801 USA; 2 Center for Bio-Integrated Electronics, Northwestern University, Evanston, IL 60208 USA; 3 Department of Biomedical Engineering, McCormick School of Engineering, Northwestern University, Evanston, IL 60208 USA; 4 State Key Laboratory of Mechanical System and Vibration, Shanghai Jiao Tong University, 200240 Shanghai, China; 5 Department of Civil and Environmental Engineering, Northwestern University, Evanston, IL 60208 USA; 6 Department of Biology, North Park University, Chicago, IL 60625 USA; 7 Department of Biology, University of Detroit Mercy, Detroit, MI 48221 USA; 8 Department of Materials Science and Engineering, McCormick School of Engineering, Northwestern University, Evanston, IL 60208 USA; 9 Feinberg School of Medicine, Northwestern University, Chicago, IL 60611 USA; 10 College of Medicine, University of Illinois at Chicago, Chicago, IL 60612 USA; 11 Department of Mechanical Engineering, McCormick School of Engineering, Northwestern University, Evanston, IL 60208 USA; 12 Department of Neurological Surgery, Feinberg School of Medicine, Northwestern University, Chicago, IL 60611 USA; 13 Department of Neurological Surgery, Ann and Robert H. Lurie Children’s Hospital of Chicago, Chicago, IL 60611 USA

**Keywords:** Sensors and biosensors, Diagnostic markers

## Abstract

Hydrocephalus is a common disorder caused by the buildup of cerebrospinal fluid (CSF) in the brain. Treatment typically involves the surgical implantation of a pressure-regulated silicone tube assembly, known as a shunt. Unfortunately, shunts have extremely high failure rates and diagnosing shunt malfunction is challenging due to a combination of vague symptoms and a lack of a convenient means to monitor flow. Here, we introduce a wireless, wearable device that enables precise measurements of CSF flow, continuously or intermittently, in hospitals, laboratories or even in home settings. The technology exploits measurements of thermal transport through near-surface layers of skin to assess flow, with a soft, flexible, and skin-conformal device that can be constructed using commercially available components. Systematic benchtop studies and numerical simulations highlight all of the key considerations. Measurements on 7 patients establish high levels of functionality, with data that reveal time dependent changes in flow associated with positional and inertial effects on the body. Taken together, the results suggest a significant advance in monitoring capabilities for patients with shunted hydrocephalus, with potential for practical use across a range of settings and circumstances, and additional utility for research purposes in studies of CSF hydrodynamics.

## Introduction

Hydrocephalus is a common and debilitating condition caused by the excess production or impaired resorption of cerebrospinal fluid (CSF) in the ventricles of the brain. Causes include congenital malformations, intracerebral hemorrhage, infection, trauma, and tumors^1^. The condition can occur in nearly all age groups from infants^1^ to elderly^2^ patients, with the latter group particularly susceptible to idiopathic normal pressure hydrocephalus (iNPH)^3^. Hydrocephalus affects over 1 million people in the United States alone. In nearly all cases, the treatment involves surgical implantation of a pressure-regulated silicone tube assembly, known as a ‘shunt’, that drains excess fluid away from the cerebral ventricles to a distal absorptive site, such as the peritoneum. Unfortunately, shunts suffer from extremely high failure rates, up to 50%^4,5^ in pediatric populations and 16% in adults^6,7^ over 6-years.

The symptoms of shunt failure are identical to those of hydrocephalus and are highly non-specific, including headaches, nausea, and drowsiness. These vague symptoms coupled with the inability of convenient, direct measurement of shunt patency frustrate straightforward diagnoses^8,9^. As a result, patients commonly undergo a range of diagnostic procedures, including evaluating the size of the cerebral ventricles with computed tomography (CT) scans or magnetic resonance imaging (MRI), evaluation for disconnection or fracture of the shunt tubing with X-ray imaging, known as a ‘shunt-series’. All such procedures represent indirect measures and they suffer from a combination of low accuracy, high costs, radiation exposure, and in the case of MRIs in pediatric patients, the need for anesthesia. More direct measures of shunt patency include nuclear medicine “shunt function” studies where a radionucleotide tracer is directly injected into the shunt system, but this procedure can be painful, and it can introduce infection into the shunt, and it is poorly suited to pediatric populations. Similarly, lumbar puncture tests also rely on hollow needles to collect spinal CSF for the assessment of pressure and viral infections. In some cases, patients are simply admitted to the hospital for long-term observation or they undergo exploratory surgeries to definitively rule out shunt malfunction. Overall, the management and treatment of patients with shunted hydrocephalus costs the U.S healthcare system over $2 billion annually, and the condition results in an impaired quality of life for both patients and their caregivers^10–12^ due to the near-constant uncertainty associated with the potential for shunt failure.

Direct, continuous measurement of CSF flow represents the most useful indicator of shunt patency^13^ and the successful development and deployment of a routine, reliable method would greatly enhance the standard of care for these patients^14–17^. Previously explored approaches range from in-line capacitive measurements^18^, to passive skin-mounted temperature sensing^19,20^. A relatively recent FDA-cleared device (ShuntCheck) uses mediated active cooling of the skin to generate thermal signals associated with flow^21–25^. The system requires, however, an ice pack for the measurement and an associated cumbersome measurement protocol that does not allow for continuous monitoring of shunt flow. Partly as a result, the findings from clinical trials of the ShuntCheck system include relatively high numbers of false positives, as patent shunts regularly experience intermittent flow^25^. Furthermore, each of these methods requires immobilization of the patient and yields only instantaneous measurements during brief examinations by trained care providers in hospital settings. The natural intermittency of CSF flow through shunts^26,27^ confounds straightforward interpretation of such instantaneous data, therefore necessitating the use of obtrusive approaches to induce flow, such as mechanical stimulation of the reservoir located in the shunt valve while the patient is immobilized ^28,29^.

Our recent work focuses on the development of a class of wearable, wireless sensor designed to address this unmet need. Here, measurements of flow follow from localized thermal actuation and sensing^30^ using a soft, thin device^31–35^ gently laminated onto the surface of the skin at the location of the shunt. The results presented in the following extend these concepts into a user-friendly, fully wireless system that enables continuous, noninvasive monitoring of CSF flow performed by patients themselves in real-world settings. Advanced designs and integration schemes exploit low-cost commercial components and flexible circuit board manufacturing techniques in optimized layouts guided by theoretical and numerical models of thermal transport and system-level mechanics. A Bluetooth Low-Energy System on a Chip (BLE-SoC) embedded system architecture allows for robust, high-quality data transfer during normal patient activities, where a miniaturized on-board, rechargeable battery supports continuous operation for several hours. On-body measurements and field trials on hydrocephalus patients (*n* = 7) reveal reliable operation during both short ‘spot-checks’ and, for the first time, extended measurements of flow during natural motions of the body and for different orientations. The results suggest broad applicability for monitoring of shunts in patients across age ranges, pathologies, and settings, including the home.

## Results and discussion

### Soft, flexible, wireless sensors for continuous flow monitoring

The basic principles of the measurement can be found elsewhere^36–39^. In the devices reported here, a miniaturized (<5 mm diameter) thermal actuator delivers small, precisely controlled thermal power (<5 mW/mm^2^) to the surface of the skin, thereby creating an imperceptible local increase in temperature (~5 K). When positioned at the location of a shunt, the directionality and the magnitude of the flow of CSF affects the resulting distribution of temperature at the surface of the skin. Specifically, the increases in temperature downstream (*T*_DS_) from the actuator are larger than those at an equal distance upstream (*T*_US_). Temperature sensors record these differences as a function of time after supplying power to the actuator. Quantitative values of flow rate can be determined from these data using multi-physics computational models that include the essential geometric parameters of the integrated system (shunt, device, and skin) and constitutive properties of the materials.

**Fig. 1:**
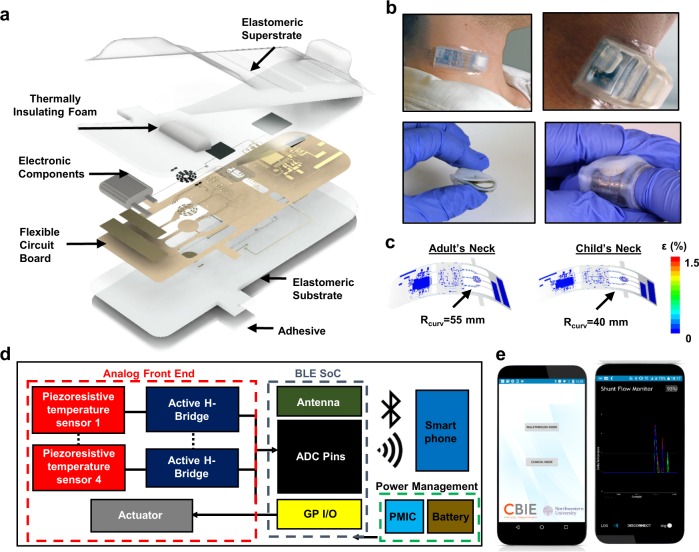
Wireless sensors for continuous monitoring of CSF hydrodynamics through shunts. **a** Exploded view illustration of the device, highlighting the flexible circuit board, electronic components, thermally insulating foam, elastomeric encapsulation and adhesive layers. **b** Optical images of devices illustrating their flexible construction and ability to mount on the skin over anatomical regions relevant to VP shunts. **c** Finite element analysis (FEA) of the distributions of strain across the fPCB during bending to a degree comparable to that required to mount on the neck region of a child (radius of curvature = 40 mm) and an adult (55 mm). **d** Schematic illustration of the electronic design for actuation, data acquisition and wireless transmission. **e** Optical image of smartphone application used for wireless readout and communication. Participants provided written informed consent to have their photos/images included as a part of this publication.

The schematic overview in Fig. 1a highlights the various design aspects, including the main elements: (i) thermal sensing and actuating components, (ii) analog front-end circuitry to convert resistance measurements of temperature into corresponding output voltages, (iii) a BLE-SoC and its associated timers and antenna to digitize and transmit these data, and also to support wireless two-way communication, (iv) power management electronics and a rechargeable lithium polymer (Li-Po) battery to supply power to the various sub-systems, (v) a flexible printed circuit board (fPCB) substrate to support and interconnect the components, and (vi) packaging and insulation layers to protect the device from the environment. The fPCB has a thickness (~115 µm) that yields low flexural rigidity (4 × 10^−4^ N-m) and sufficient degrees of flexibility to conform and bond to the curved surface of the skin with a mild adhesive where the shunt is most superficial, typically near the neck or the clavicle. This mechanics follows from an island-bridge configuration, designed to localize bending strains to the interconnected structures and away from the electronic components. The result (i) facilitates conformal contact with the skin while reducing potential for delamination and (ii) minimizes strain on the rigid electronic components and soldered interfaces between the components and the fPCB. These effects are apparent in Fig. 1c, where mechanical finite element analysis (FEA) results indicate that the strains on the interconnect layer remain low (<1%) during routine bending associated with mounting on the neck for children (radius of curvature ~40 mm) and adults (55 mm).

The planar areas support electronic components that connect through thin, mechanically stable conductive traces on the fPCB. A soft, low-modulus silicone elastomer (E ~ 60 kPa) encapsulates the entire system (Fig. 1b). Several considerations inform the choice of mechanics, materials, and form factor, including adhesion, comfort, safety, and thermal transport. A thin, soft silicone layer (100 µm thickness, *E* = 1.4 MPa) on the underside of the device completely covers the skin-facing side of the flexible circuit board. The ability to establish strong, yet repeatable and non-irritating contact with the skin represents a key consideration, facilitated by the thin, soft construction of the device and by a medical-grade, double-sided acrylate-silicone adhesive. The high adhesion energy of the acrylate layer (~350 N/m)^40^ establishes strong contact to the device, while the comparatively low adhesion of the silicone layer (~33 N/m) forms a gentle interface to the skin while maintaing excellent thermal coupling. A liner-release layer with a custom laser-structured tab facilitates handling and mounting. Peeling back the tab exposes the adhesive for mounting on the skin. The entire silicone and double-sided adhesive assembly has a thickness of 120 µm and adds a thermal mass of 14 mJ/cm^2^-K, equivalent to that of a <50 µm thick layer of skin.

Figure 1d presents a schematic illustration of the operation of the system. A custom software application (Fig. 1e) serves as a control interface, as well as a means to record, store and display data on any BLE-enabled device (smartphone, tablet, etc.). The software also provides step-by-step on-screen instructions to guide users on the operating procedures. A toggle-switch on the user interface controls the operation of a resistive thermal actuator, with power provided through the BLE-SoC. An analog front-end circuit based on an active Wheatstone bridge network (Supplementary Fig. 1) converts data from resistive temperature sensing elements into voltages. The BLE-SoC digitizes and transmits these data to the smartphone, where they can be analyzed to yield rates of CSF flow.

**Fig. 2:**
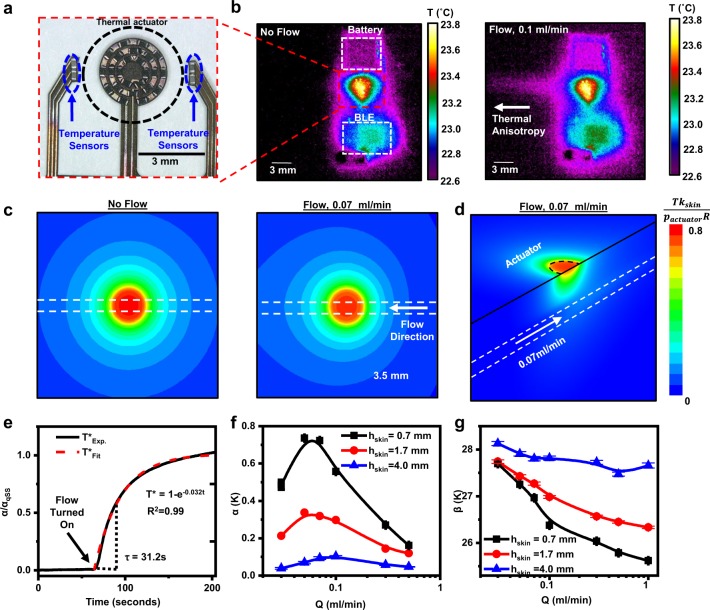
Thermal and mechanical characterization of the flow sensor. **a** Optical micrograph of the circular thermal actuator and sensing elements on fPCB. **b** Infrared (IR) images of thermal actuation in the absence (left) and presence (right) of flow (*Q* = 0.07 ml/min) in benchtop shunt assembly. **c** Temperature distributions generated by 3D finite element analysis (FEA)(top-view), illustrating the effects of no flow (left) and flow (right) for a representative case, with color scheme corresponding to a normalized change in temperature. **d** Cutaway view of images generated by 3D FEA illustrating heat transfer through near-surface skin layers for *Q* = 0.07 ml/min. **e** Plot of *α/α*_qss_ = *(T*_DS_ − *T*_US_*)/ (T*_DS_ − *T*_US_*)*_qss_ as a function of time before and after a step-change in flow, from 0 ml/min to 0.3 ml/min, at *t* = 65 s, for the shunt phantom system with an equivalent skin thickness of 1.7 mm and fitted with an exponential form to yield a time constant of 31 s to reach 63.7% of a quasi-steady-state value $\left( {\frac{{\partial \alpha (t)}}{{\partial t}}\sim 0} \right)$ **f** Plot of α as function of different physiologically relevant flow rates (0.03 < *Q* < 0.5 ml/min) and skin thickness, *h*_skin_ = 0.7 mm (black), 1.7 mm (red) and 4.0 mm (blue), highlighting non-monotonic behavior and an inflection point at *Q* = 0.07 ml/min. **g** *β* *=* (*T*_DS_ + *T*_US_)*/2* computed for the same flow rates and skin thicknesses as in **f**. Error bars in **f** and **g** correspond to standard deviations over 100 s for a single experiment.

The actuation and sensing components represent the most critical elements of the device. Negative temperature coefficient (NTC) temperature sensors (image in Fig. 2a) provide high accuracy and precision in measurements of temperature (<5 mK) (Supplementary Fig. 2), with minimal hysteresis, good stability, and negligible drift. (Supplementary Fig. 3). For redundancy, the device incorporates a pair of NTC elements upstream and downstream, for a total of four NTCs, located 1.5 mm from the edge of the actuator, as seen in Fig. 2a. The measurement involves delivery of thermal powers of 2–5 mW/mm^2^ to the skin. The actuator exploits 24 surface-mount resistors (300 µm × 250 µm × 600 µm) arranged in a dense, circular array (Fig. 2a) over an area of 7.0 mm^2^, to produce spatially uniform heating with a magnitude controlled by the applied voltage. As an example, a voltage of 3.3 V applied to an actuator constructed with 20 Ω resistors, for a total resistance of 24 × 20 Ω = 480 Ω, results in a current of *I* = 7 mA and a power of *P* = 23 mW, over an area of 7 mm^2^ to yield a power density of 3.3 mW/mm^2^. The result is a temperature increase of <5 K uniformly over the area of the actuator when mounted on skin or on a benchtop shunt phantom system, as confirmed by IR thermographs in Fig. 2b.

In the absence of flow, heat diffuses isotropically from the actuator. The presence of flow skews the temperature distribution in the direction of the flow, visible as a characteristic ‘tail’ in Fig. 2b (right). Increased actuation power results in an improved signal to noise ratio (SNR), but also in higher local temperatures (Supplementary Fig. 9). A power density of <6 mW/mm^2^ represents a tradeoff between maximizing SNR and maintaining the maximum increase in temperature below biologically acceptable limits (<12 K) with a factor of safety, as discussed in a subsequent section. Three-dimensional (3D) FEA yields distributions of temperature (Fig. 2c, d, Supplementary Fig. 6) that qualitatively match those determined by IR imaging for the phantom system. These models use geometrical and constitutive properties associated with the system, such as the skin thermal conductivity (*k*_skin_), diffusivity (*ν*_skin_), thickness (*h*_skin_), flow rate (*Q*), and others, in the form of lumped, nondimensional quantities that inform device architecture and processing algorithms, as reported previously^38^. Separate studies by IR thermography reveal that the heating associated with other components of the system such as the battery, voltage regulator, and BLE-SoC is negligible.

The addition of a thermally insulating polyurethane foam over the sensing and actuating elements improves the SNR by an order of magnitude (Fig. S10) and is a fundamentally enabling aspect of the design reported here by significantly reducing sources of noise that can be induced by air flow. This effect can be understood by considering the rate of heat transfer from the NTC to its surroundings by free-convection and its linear dependence on the convective heat transfer coefficient, *H*_free_. The side-wall of the NTC can be modeled as a simple vertical plate, and accordingly, *H*_free_ is^41^

1 $$H_{{\mathrm{free}}} = \frac{{k_{{\mathrm{air}}}CRa_L^n}}{L}$$

where *L* is the height of the NTC element (~300 μm), *n* and *C* are empirical fitting factors known to be 0.25 and 0.59 respectively for laminar flows^41^ and *Ra*_*L*_ is the Rayleigh number for free convection across *L*, given by

2 $$Ra_L = \frac{{g\beta (T_{NTC} - T_\infty )L^3}}{{\nu \varsigma }}$$

where *g* is the acceleration due to gravity, *β* is the volumetric fluid expansion coefficient of air, *ν* is the thermal diffusivity of air and *ς* is the kinematic viscosity of air. *T*_*NTC*_ and *T*_*∞*_ are the temperature of the NTC and its surroundings, respectively. The addition of a foam layer effectively prevents air circulation around the NTC, and therefore free-convection effects across the vertical surface of the NTC, with a magnitude that is only weakly dependent on foam formulation and pore size, for materials examined here, above a critical thickness of ~1 mm (Supplementary Fig. 10).

To quantify flow-induced thermal anisotropy, consider the parameter $\alpha \equiv (T_{DS} - T_{US})$ as the difference between the average temperature determined by the two downstream and the two upstream NTC sensors, where *T*_US_ and *T*_DS_ represent changes in temperature from a steady-state baseline value prior to actuation. In the absence of flow, *α* is ~0 K and in its presence, *α* > 0, with a value that is typically >30 times larger than the noise for practical scenarios of relevance to hydrocephalus patients, as shown in Fig. 2e. The temporal response of α to a change in flow is a function of the thermal mass of the fPCB assembly, including the NTCs and the actuator, and the characteristic diffusion time associated with thermal transport through underlying skin to the shunt, *t*_diffusion_ ~ *h*_skin_^*2*^*/ν*_skin_. The case of a step-change in flow on a shunt phantom system from 0 ml/min to 0.3 ml/min for *h*_skin_ = 1.5 mm is in Fig. 2e. At time *t* = 65 s, the flow activates, resulting in a transient temperature rise that can be fit to the form *α(t)/α*_qss_ = *1−exp*(*−Kt*), where *α*_qss_ is a quasi-steady-state value of *α* such that $\left( {\frac{{\partial \alpha (t)}}{{\partial t}}} \right)_{\alpha \;=\; \alpha _{{\mathrm{qss}}}}\sim 0$ and *K* is a fitting constant found to be 0.032 s^−1^. This relationship suggests that the first 100 s after actuation of the measured *α(t)* curve can be treated as a transient period, after which temperature reaches ~95% of its steady-state value.

As discussed previously^30,38^, *α* varies non-monotonically with flow rate, as shown in Fig. 2f, with a peak sensitivity at 0.07–0.1 ml/min for the designs reported here. High sensitivity extends across a physiologically relevant range of flow rates, i.e. 0–0.5 ml/min^27,42–44^. The thickness of the skin and/or underlying subcutaneous fat layers that lies over the shunt, *h*_skin_, also strongly influences *α*. As *h*_skin_ increases, the sensitivity decreases. Though the sensitivity is insufficient for *h*_skin_ larger than ~4 mm, this limitation is not expected to be relevant for measurements over the neck/clavicle region where *h*_skin_ is typically between 0.5 mm and 2 mm. To distinguish between flow rates associated with identical values of *α* on either side of its peak value, a second parameter, $\beta \equiv (T_{{\mathrm{DS}}} + T_{{\mathrm{US}}}){\mathrm{/}}2$, the average change in temperature must be considered. *T*_DS_ varies non-monotonically with flow, increasing with flow for 0 < Q < 0.07 ml/min and decreasing with flow for *Q* > 0.07 ml/min. By contrast, *T*_*US*_ decreases with flow for a full range of flow rates. As a result, their average, *β*, is relatively constant at low flow rates and decreases with flow at high rates for steady-state measurements. In effect, *β* is a measure of the increased net thermal transport (i.e, non-directional) due to convection, and decreases monotonically with flow rate (Fig. 2g). As a result, *β* can yield information about flow regime (high vs. low) while *α* can serve as a measure of flow rate. Combining these two parameters allows for the determination of flow rate, as shown experimentally in a subsequent section. These benchtop measurements also reveal the tolerance associated with rotational misplacement of the sensor (up to 45°, Supplementary Fig. 7) as well as its translational misplacement (up to 5 mm, Supplementary Fig. 8).

### On-body flow monitoring and imaging

**Fig. 3:**
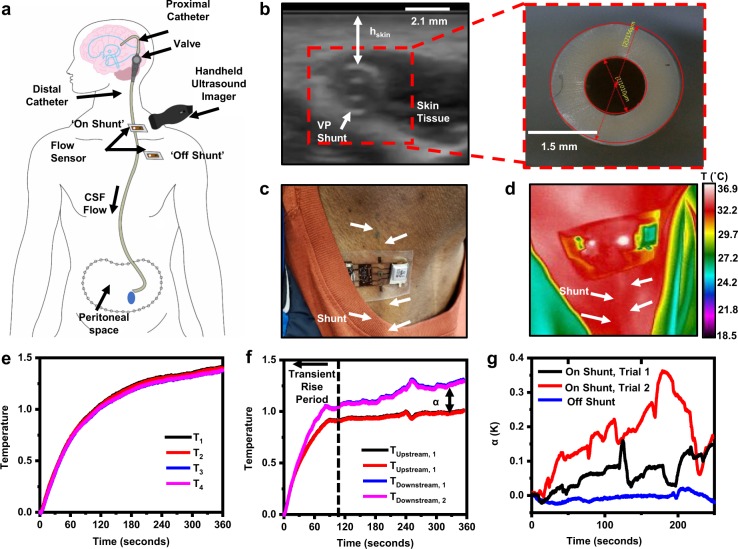
On-body ultrasound, IR and device measurements on a healthy outpatient (M, 21) with a VP shunt. **a** Schematic illustration highlighting on-shunt, off-shunt and ultrasound measurement locations. **b** Ultrasound image of VP shunt at location 2 cm distal to clavicle (left), with optical micrograph of unimplanted shunt (Medtronic, Bactiseal) for scale (right). **c** Optical image of device mounted on skin over shunt. **d** IR thermograph of operational device, illustrating a maximum local temperature rise at the thermal actuator of 4.7 °C, with outline of shunt visible downstream of actuator. **e** Raw temperature recorded from temperature sensors at off-shunt measurement at base of pectoral muscle distal to clavicle. **f** Raw temperature measurement from four temperature sensors at on-shunt measurement site, showing difference between downstream and upstream temperature signals, *α*. **g** *α* computed for two separate trials on the same patient over the shunt and one trial at off-shunt location. Participants provided written informed consent to have their photos/images included as a part of this publication.

Measurements on human subjects involve placement of the device at a distal location along the shunt above the clavicle (referred to as on-shunt), guided by visual examination and tactile feel. Alignment marks on the device and temporary markings on the skin, formed with a surgical pen, facilitate alignment and positioning. Often the shunt is clearly visible (as in Fig. 3c), but it is always straightforward to palpate, especially over the clavicle. An additional measurement at a location of the skin adjacent to the shunt but devoid of near-surface vasculature (referred to as off-shunt) serves as a control, representing the ‘zero-flow’ case (Fig. 3a). A handheld ultrasound instrument yields images of the skin and the underlying shunt tubing (Butterfly IQ, CT, USA), as illustrated in Fig. 3b for the case of an asymptomatic adult male volunteer (M, 21). The distance from the top outer surface of the shunt tubing to the surface of the skin in this case is 1.4 mm (Fig. 3c). Locations where the shunt is easily palpated are typically <2 mm from the surface of the skin (additional ultrasound images and measurements are in Supplementary Fig. 12).

Operating the actuator at a fixed, power-regulated level (4 mW/mm^2^) results in a local temperature rise of <5 K at the surface of the skin (Fig. 3d, Supplementary Fig. 13). As with the results with the benchtop system in Fig. 2b, IR imaging reveals the outline of the shunt after thermal actuation (Fig. 3d), with temperatures that gradually decrease with increasing distance from the thermal actuator. Linear temperature profiles at upstream and downstream locations in orientations perpendicular to the flow of the shunt (Supplementary Fig. 13) reveal the effects of the actuator in raising the local temperature of the CSF, and of the flow, in transporting the heat anisotropically downstream. At downstream locations, the elevated temperature of the CSF results in a temperature profile that reaches a maximum over the shunt, where the center of the shunt equilibrates at a temperature ~0.4 K above its surroundings. Similar measurements at upstream locations show negligible variations in temperature, as expected.

Results of IR imaging also allow for evaluations of thresholds for safe levels of heating of the skin over time periods relevant to the measurement. Here, the cumulative equivalent minutes of thermal exposure at 43 °C (CEM_43_) serves as a useful metric^45,46^ as defined as

3 $$CEM_{43}=tR^{(43-T)}$$

where *t* is the total heating time in minutes, *T* is the local skin temperature in °C, and *R* is a scaling factor, empirically known to be 0.25. The 43 °C inflection point is associated with cellular breakdown and *CEM*_*43*_ values of >400 are associated with irreversible thermal damage to skin^46^. IR measurements over the region of maximum increase in temperature at the actuator reveals values of ~36.5 °C (Supplementary Fig. 13), corresponding to a *CEM*_43_ of ~10^−3^, over a 5 min measurement period, several orders of magnitude below the threshold for damage. Even for long measurement times of ~6 h (representing the total battery life of the device) the value of *CEM*_43_ is quite small (~0.04), thus demonstrating that the risks are minimal even for long-term continuous monitoring. In all studies discussed here, patients could not sense increases in temperature caused by the actuator.

In the absence of flow, or at a suitable ‘off-shunt’ location such as the base of the pectoral muscle immediately distal to the clavicle, measurements from the temperature sensors increase smoothly and monotonically with values that are nearly the same to within 50 mK (Fig. 3e) resulting in root-mean square (RMS) values of *α*_RMS_ ~ 13 mK with peak-to-peak variations of 8 mK, across a 100 s averaging window (representing the thermal response time) (Fig. 3g). Similar measurements over the shunt display clear thermal anisotropy (Fig. 3f), with *α*_RMS_ ~ 250 mK (75 mK) and peak-to-peak variations of 60 mK (25 mK) for high flow (low flow) cases (Fig. 3g). These data establish that values of *α* > 50 mK are above the noise level and can be assumed to result from flow. The observations also reveal that the transient period of the response has a duration of ~100 s. The studies reported here focus only on the quasi-steady-state response, defined by operation after this transient period.

### Instantaneous “spot-checks” of shunt patency

**Fig. 4:**
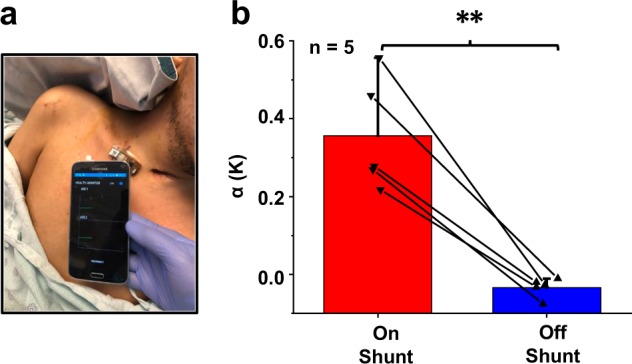
Short “spot-check” measurements on in-patients with confirmed flow. **a** Optical image of device mounted on skin location overlying shunt, with smartphone for continuous data readout. **b** Mean of α computed for *n* = 5 patients with confirmed flow, or who are asymptomatic, over on-shunt and off-shunt locations. (**p < 0.01 for paired student-*t* test). Error bars correspond to standard deviations across five subjects. Participants provided written informed consent to have their photos/images included as a part of this publication.

Evaluations on additional patients establish the repeatability and robustness of operation across a range of age groups and pathologies without suspected shunt malfunction. An image of a device placed on the clavicle of an otherwise healthy outpatient (M, 21) is in Fig. 4a, where surgical markings outline the location of the shunt. A smartphone with the software application operated by an attending or resident physician receives temperature data from the device and plots the results in real-time on a graphical user interface. The wireless interface allows the physician freedom of motion, within a range of ~6 m from the bed of the patient, without loss of connection or aberrant signals. Similarly, the patients can also move freely in and around their hospital bed without disruption in wireless connectivity or motion-induced artifacts in the measurement (further images in Supplementary Figure 14). In all cases, on-shunt measurements averaged over 100 s after the transient 100 s period (*α*_on shunt_ = 0.35 ± 0.14 K) differ (*p* = 0.003) from off-shunt measurements (*α*_off shunt_ = −0.03 ± 0.02 K) in a paired student-*t* test (Fig. 4b). These results represent significant improvements over those obtained with our previously reported devices^30^, largely because (i) the wireless embodiment introduced here minimizes motion-induced partial delamination and strain-induced electrical noise and (ii) the insulating foam isolates the measurement system from ambient thermal fluctuations and time-variant convective effects.

### Continuous flow monitoring

**Fig. 5:**
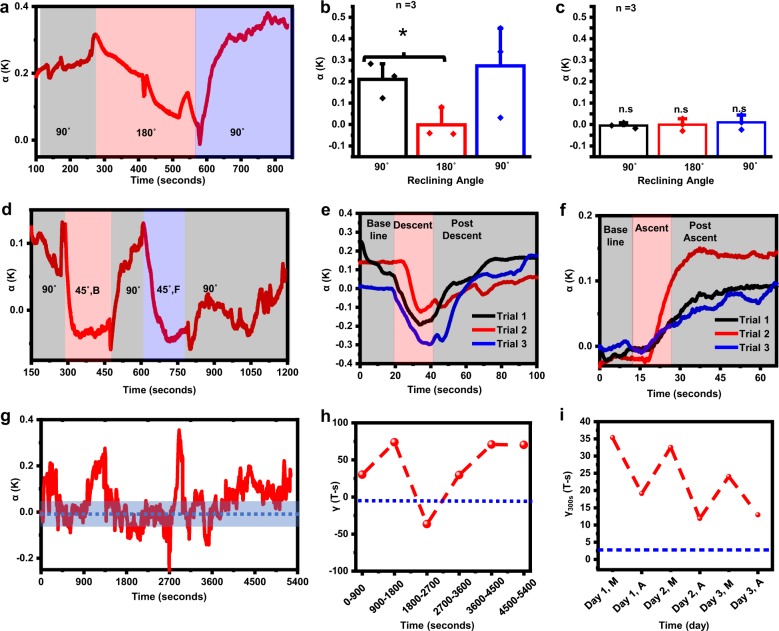
Advanced measurements of continuous CSF hydrodynamics on healthy out-patients. **a** *α* computed for a healthy outpatient (Male, 21) at three positions, sitting up at 90°(grey shaded area), supine at 180°(red shaded area) and sitting up at 90° (blue shaded area). **b** *α* averaged over a 100 s window for the three positions, across *n* = 3 patients at on-shunt locations. **c** Same as **b**. but measured at off-shunt locations. **d** *α* for a volunteer (F,16) leaning forwards (F) and backwards (B) at 45°, and sitting up at 90°, respectively. **e** *α* computed for continuous measurement over on-shunt location as volunteer (Male, 21) outpatient descended on elevator moving at 1.5 m/s across an elevation of 17 m. **f** Same as **e** but measured while same volunteer ascended on elevator, with baseline adjusted to same starting value of α. **g** *α* computed for continuous 1.5-h measurement on volunteer (Male, 21) during normal activities, showing intermittent flow. **h** Area-under curve, *γ*, computed for *α*(**t**) over different sampling windows for data in **g** as a measure of total flow output during fixed intervals. **i** *γ* computed over 5-min sampling window for same patient across three days, during morning (M) and afternoon (A) measurements showcasing variability in flow patterns across time of day. Error bars in **b** and **c** correspond to standard deviations across three subjects.

Continuous flow measurements on freely moving patients represents a fundamentally new mode for monitoring. Here, the utility of the device reaches beyond performing simple binary assessments of flow/no-flow to establishing correlates between dynamic, real-time changes and patient sensations/activities. For example, changes in body orientation can affect flow, as suggested by patient complaints of headaches when lying down or immediately after standing up. In a healthy, asymptomatic outpatient, (M, 21), measurements before, during, and after changes in body orientation suggest corresponding changes in flow (Fig. 5a). Initially, measurements performed with the patient sitting upright (90°) yield values consistent with a normal, healthy daytime flow at a constant rate (*α* ~ 0.2–0.3 K). Reclining to a supine position (180°) leads to a gradual decline of flow over ~200 s, consistent with previous findings performed with externalized drains^44^ and in-dwelling shunts with flows measured using radioactive tracers^47^ and ultra-sound imaging with air-bubbles introduced into the shunt^20,26^, until the volunteer returns to a sitting upright position (90°), where the measurements indicate a return to baseline flow rates. Additional tests on two healthy, asymptomatic outpatients (F, 15, M, 27) indicate similar results (Fig. 5b, raw data in Supplementary Fig. 15) with clear (*p* = 0.04) differences between measurements during an initial upright period and a subsequent supine period as computed for a paired t-test. Control measurements off-shunt show no thermal anisotropy, independent of body orientation, as expected (Fig. 5c).

Patients can often identify instances of aberrant flow based on the onset of characteristic headaches. In one case, an otherwise healthy asymptomatic outpatient (F, 16) described changes in position from upright to leaning forward (45°) as a case for headaches, for example, during reading. Results from continuous monitoring on this patient (Fig. 5d) show that leaning both forward and backward (45°) leads to an instantaneous and significant reduction in flow that coincided with headaches. Reversal of flow, as indicated by a negative value of α, can also occur. In both cases, flow appears to recover to a positive, baseline value, though at different rates. In another instance, an otherwise healthy, outpatient (M, 21) complained of headaches during rapid inertial changes associated with riding on elevators in high-rise buildings. Measurements on this patient during three routine elevator ascents and descents reveal characteristics consistent with corresponding changes in flow during the periods of acceleration, where descent reduces flow, to the level of back-flow (Fig. 5d), and ascent enhances flow (Fig. 5f).

In addition to these episodic changes, variations can be measured over longer times, either through continuous monitoring or by comparison of repeated measurements. Continuous monitoring of an outpatient (M, 21) for a period of ~1.5 h, during normal behaviors, with the acquisition smartphone placed in a pocket, illustrates these capabilities. These data (Fig. 5g) demonstrate intermittent flow with time scales (~20 min) that are consistent with prior data collected on patients with externalized ventricular drains^48^. Total CSF volume extracted during shunt taps serves as an important diagnostic measure of patient health, and time integration of *α(t)* yields a parameter, *γ*, as a correlate for total volumetric flow during a fixed time interval. Such integrated measurements for 15-min time intervals highlight changes in flow output over the monitoring period (Fig. 5h) as an indicator of shunt intermittency. On longer time-scales, *γ* values can also serve as points of comparison across days or longer (Fig. 5i).

### Conversion of thermal anisotropy to quantitative flow rate

**Fig. 6:**
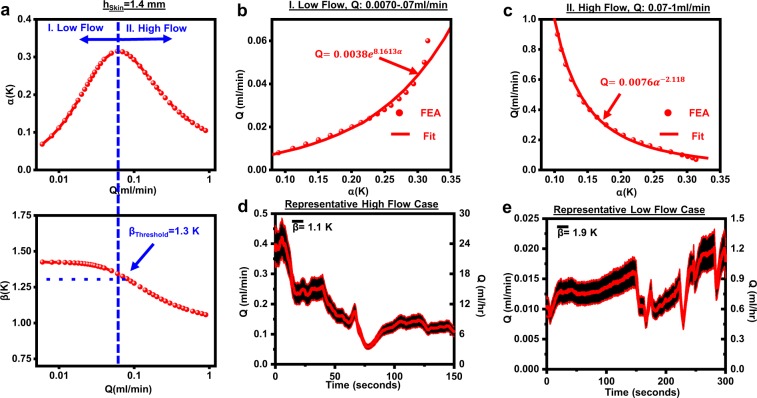
Flow rate measurements on a healthy out-patient (M, 21). **a** FEA-simulated curves for *α(Q)* (top) and *β(Q)* (bottom) for *h*_skin_ = 1.4 mm, corresponding to measured value on the out-patient, with an inflection point at *Q* = 0.07 ml/min corresponding to *β* = 1.3 K. **b** Simulated data (circles) and fit (line, exponential curve) for low-flow regime corresponding to 0.007 < *Q* < 0.07 ml/min. **c** Simulated data (circles) and fit (line, power-law curve) for high-flow regime corresponding to 0.07 < *Q* < 1 ml/min. **d** Representative continuous high-flow measurements on the out-patient with shaded regions representing uncertainty estimates (±15%). **e** Representative low-flow measurements on the out-patient with shaded regions representing uncertainty estimates (±15%).

The real-time measurement of a quantitative value of the flow rate, beyond the metric *γ*, represents a key capability. Accounting for *h*_skin_ (1.4 mm for the outpatient discussed here) via ultrasound imaging allows for 3D FEA models for *α(Q)* and *β(Q)*, across a range of physiologically relevant CSF flow rates from 0.007 to 1 ml/min (0.4–60 ml/hr) (Fig. 6a). Other parameters in the model include the thermal conductivity (*k*_skin_ = 0.3 W/m-K^35^), density (*ρ*_skin_ = 1050 Kg/m^3^ ^49^) and heat capacity (*C*_p,skin_ = 3500 J/Kg-K^35^) of the skin, and corresponding properties for the shunt (*k*_shunt_ = 0.21 W/m-K, *ρ*_shunt_ = 965 Kg/m^3^, *C*_p,shunt_ = 1460 J/Kg-K^50^). While the constitutive and geometrical properties of the shunt are largely known and fixed, *h*_skin_, *k*_skin_, *ρ*_skin_, and *C*_p,skin_ can vary from patient to patient, across a well-characterized^35,51^ range. The effects of each of these parameters are discussed elsewhere^30^. Values of *α* and *β* computed via 3D FEA are within acceptable levels of agreement (~15%) with measured values on a benchtop shunt system (Supplementary Fig. 16) configured to approximate the relevant anatomy (*h*_skin_ = 1.7 mm). The inflection point between high and low flow regimes is 0.07 ml/min, corresponding to *β* = 1.3 K, as shown in Fig. 6a. Dividing *α(Q)* into low-flow and high-flow components and fitting each separately allows for conversion from *α* to *Q*. The low-flow regime is fitted via an exponential relationship (*Q* = 0.0038e^8.161*α*^*)* (Fig. 6b) while the high-flow regime is fitted via a power law (*Q* = 0.007α^−2^^.12^) (Fig. 6c). The fits are in strong agreement with FEA models for 0.01 K < *α* < 0.5 K. Representative high and low-flow cases computed in this manner, with their corresponding values of *β* are in Fig. 6c, d, where the shaded regions correspond to uncertainty estimates (±15%) inherent to the fitting.

The results of 12 spot-check measurements performed on the same healthy outpatient over a 3-day period are in Table 1 while sitting or standing upright. Averaging over 100 s windows yields values for *β* to define the high and low-flow regimes. Averaged values of *α* over the same window yield values for *Q* via the appropriate conversion equation, with standard deviations of ±15% associated with fitting uncertainties as the upper and lower bounds. The highest and lowest flow rates over the 3-day measurement are 0.26 ± 0.05 ml/min (15.6 ± 3 ml/h) and <0.01 ml/min (0.6 ml/h), respectively. Across the 12-measurements, four instances of high flow (*Q* > 0.07 ml/min, β < 1.3 K), six instances of low-flow (*Q* < 0.07 ml/min, *β* > 1.3 K), and two instances of transition-flow (*Q* ~ 0.07 ml/min, *β* ~ 1.3 K) occurred. The average value of *Q* across all measurements was 0.08 ± 0.07 ml/min (4.8 ± 4.2 ml/h). These flow rates correspond well to established values for pediatric and adult patients, across several studies on externalized drains^44,48^ and indwelling shunts^20,26,27,47^. Flow varies significantly over a 60-min period, consistent with current understanding of CSF hydrodynamics^20^, and extended measurements on the same patient (Supplementary Fig. 17). These data suggest the importance of either a single continuous measurement or several short measurements over a 60-min period to accurately capture flow characteristics. The timing of the measurements (late morning to early evening) could be an additional important factor^26^, and studies on healthy patients during sleep and early-morning periods represent the basis of ongoing work. Ongoing studies on externalized drains will serve as a means to calibrate sensor measurements against drainage volumes over fixed time intervals.

**Table 1:** Flow rates calculated for 12 spot check measurements on healthy out-patient (M,21) across 3-day period, with values of *β* for classification into high (*β* < 1.3 K green), low (*β* > 1.3 K, red*)* and transition (*β* ~ 1.3 K blue) flow regimes.

Day	Time of Measurement	*β* (K)	Classification	Flow rate (ml/min)
1	12:57 PM	1.12 ± 0.02	High Flow	0.14 ± 0.03
1	1:17 PM	1.74 ± 0.04	Low Flow	0.01 ± 0.002
1	1:46 PM	1.48 ± 0.03	Low Flow	<0.01
1	2:01 PM	1.64 ± 0.02	Low Flow	0.02 ± 0.007
1	2:45 PM	1.70 ± 0.02	Low Flow	0.05 ± 0.02
2	1:36 PM	1.12 ± 0.01	High Flow	0.12 ± 0.04
2	3:20 PM	1.00 ± 0.04	High Flow	0.20 ± 0.04
2	4:08 PM	1.65 ± 0.01	Low Flow	0.01 ± 0.003
2	5:00 PM	1.11 ± 0.04	High Flow	0.26 ± 0.05
3	10:57 AM	1.40 ± 0.03	Transition	0.06 ± 0.01
3	11:30 AM	1.72 ± 0.01	Low Flow	0.01 ± 0.002
3	1:30 PM	1.37 ± 0.01	Transition	0.08 ± 0.01

All data are averaged over 100 s measurement windows, and uncertainty estimates represent standard deviations over 100 s combined with 15% fitting uncertainty.

The technology reported here represents a significant engineering extension of our previously published platfoms^30^, combining a collection of concepts in sensor design, thermal engineering, flexible circuit board manufacture, wireless data transmission, and digital health to yield the first continuous CSF flow monitor for patients with hydrocephalus. This type of sensor creates important opportunities for the treatment and care of patients with hydrocephalus, both for clinical/in-patient diagnostics and for research into CSF hydrodynamics. The ability to correlate flow patterns with patient observations suggests a pathway to the individualized care of these patients, in a way that can be translated to at-home settings. This platform will also allow continuous monitoring of patients during normal activities (sleep, exercise, etc.) and during shunt malfunction, to yield insights into shunt-behavior, to allow for an improved understanding of the condition, and to enable enhancements in shunt designs. Future studies will focus on statistical validation in a larger patient population through appropriately powered studies to establish values for positive and negative predictive value for shunt malfunction.

## Methods

### Fabrication and assembly of electronics

Fabrication of the sensor and supporting electronics began with processing of a trilayer film of copper/PI/copper (18 µm/75 µm/18 µm, Pyralux, DuPont Inc.) with a UV laser cutter (LPKF U4) to pattern traces, bond pads, and unplated vias. Successive washes in stainless steel flux (Worthington Inc), deionized water, and isopropanol (Fisher Scientific) removed surface oxides and prepared the resulting flexible PCB (fPCB) for assembly. Reflow soldering with low-temperature solder paste (TS391LT, ChipQuik) established electrical contacts between commercial-off-the-shelf components (microcontroller, operational amplifier, dropout regulator, switch, battery, bridge/tuning resistors, heating/actuation components, matching capacitors, and other supporting components) and the fPCB. Ultra-thin flexible wire (36 AWG Copper Stranded Wire, Calmont Inc.) connected the via holes between the top and bottom layers of the fPCB.

### Encapsulation and skin adhesive

Tri-axis milling with a CNC machine (MDX 540, Roland) formed male and female aluminum molds. Spacers between the male and female molds defined the thicknesses (~0.5 mm) of silicone shells as enclosures for the devices. Pouring and casting a medical-grade liquid silicone prepolymer and curing agent (Silbione 4220 Elkem) into the female mold, followed by aligning and compressing the male mold with a force of ~5 N while curing at 100 °C for 15 min formed a shell. Separately, spin-casting (3000 rpm for 60 s) (poly)methyl methacrylate (PMMA) followed by baking (180 °C for 180 s) formed a solid film (thickness ~700 nm) on a clean glass slide to create a hydrophobic surface. Spin-casting (1000 rpm for 60 s) and curing (70 °C for 8 min) a (poly)dimethylsiloxane (PDMS) silicone prepolymer (Sylgard 184, 10:1, Dow Chemicals) onto this layer of PMMA formed a thin (100 µm), partially cured film of silicone. Mounting the fPCB on this layer and curing at 100 °C for 15 min established a mechanical bond between the two materials. Drop-casting an uncured, liquid prepolymer of (poly)urethane foam (FlexFoam, Smooth-On Inc.) onto the sensing and actuating components and curing at 50 °C for 60 min created a 2–3 mm- thick, solid layer of foam for thermal insulation. Drop-casting a liquid PDMS (Sylgard 184, 10:1) prepolymer along the outline of the fPCB, followed by aligning and mounting the shell and curing at 100 °C for 15 min bonded the shell to the underlying silicone substrate, to seal the device. The hydrophobic PMMA surface facilitated easy removal from the glass slide by careful peeling. Finally, laser cutting formed a clean outline for the device, thereby completing the encapsulation process.

Separately, laser structuring formed the outline of a commercially available medical-grade adhesive (3M 2477P) with a double-sided silicone-acrylate construction and liner layers. Peeling back the liner on the acrylate layer (adhesion energy 350 N/m) and exposing the surface to a UV-O lamp (Jelight Inc.,) created a hydrophilic surface. Mounting this layer on the bottom silicone of the fPCB completed the device assembly. Peeling back the liner material on the skin-facing silicone adhesive prior to patient application allowed for strong, non-irritating adhesion to skin (adhesion energy 35 N/m).

### Benchtop experiments with phantom skin model

A benchtop model system allowed for simulation of the flow rates, skin thicknesses and skin thermal properties relevant to measurements of CSF flow through shunts. The system comprised the distal catheter (OD = 2.1 mm, ID = 1.1 mm) of a VP shunt assembly (Medtronic Ares, Medtronic Inc.,) embedded inside a silicone skin phantom, at a depth of 1.1 mm. A well-mixed combination of polydimethylsiloxane (PDMS) (Sylgard 184, 10:1, k_Syl184_ = 0.21 w/m-K) and PDMS doped with carbon black microparticles (Sylgard 170, 1:1 k_Syl 170_ = 0.48 W/m-K) served as skin phantoms. Different ratios of the two materials yielded a range values of k_skin_^36^ relevant to the stratum corneum (*k*_SC_ ~ 0.25 W/m-K), epidermis (*k*_epidermis_ ~ 0.35 W/m-K) and subcutaneous fat layer (*k*_Fat_ ~ 0.2 W/m-K). Spin-casting and laminating silicone sheets of different thicknesses (0.5, 1.0, 2.0, 3.0, 4.0 mm) and mixing ratios onto the phantom assembly simulated the desired values of *h*_*skin*_ and *k*_skin_. A calibrated syringe pump (PicoPlus Elite, Harvard Apparatus) connected to the distal catheter through a VP shunt valve (Medtronic Strata, Medtronic, Inc.,) supplied flow through the assembly. Water served as the test fluid, as it forms 99% of CSF^52^. A commercially available medical-grade adhesive (2477 P, 3 M Inc., described above) bonded the device to the phantom assembly.

Experimental validation followed two protocols. Protocol 1 simulated step-changes in flow to measure real-time sensitivity and time dynamics. Mounting the device on the skin phantom assembly with no flow (i.e, 0 ml/min) allowed it to thermally equilibrate with the surface temperature for 120 s. Following this equilibration period, operation of the actuator resulted in a local increase in temperature of <5 K over 180 s. Flow began at *t* = 180 s after actuation, for an additional 180 s. At *t* = 360 s after actuation, flow ended, and the temperatures re-equilibrated for a final 180 s. Conducting this experiment for two flows rates, 0.05 and 0.5 ml/min, bounded the responses expected for the full range of healthy flow rates. The time intervals used in these tests exceeded the natural response times of the device, syringe pump and skin phantom.

Protocol 2 simulated constant flow during a 5-min measurement period, to establish values of *T*_DS_, *T*_US_, *α* and *β* for steady-state flow conditions, as a scenario of direct relevance to on-body patient trials. Here, flow at a pre-determined rate initially equilibrated for a period of 60 s. Laminating the device onto the skin phantom over the shunt for 120 s allowed the system to thermally equilibrate, as in protocol 1. Operating the actuator and collecting data from the 4 NTCs for a period of 300 s completed the protocol. We conducted this protocol for the following flow rates: *Q* = 0, 0.03, 0.05, 0.07, 0.1, 0.2, 0.3, 0.5, 0.7 ml/min. Temperature calibrations (Supplementary Fig. 1) allowed conversion of changes in resistances of the NTCs to temperature measurements^30^.

### On-body trials

On-body patient and volunteer studies (IRB Protocol STU0020542, Northwestern Memorial Hospital, IRB Protocol 2018-1672, Lurie Children’s Hospital) began with palpating the shunt through the neck or clavicle to identify the location of the superficial shunt. Where available, a handheld ultrasound imaging system (iQ, Butterfly Inc., CT, USA) facilitated location of the shunt and enabled measurements of h_skin_. Cleaning the skin with an alcohol wipe and marking the shunt with a surgical pen prepared the skin for the measurement. Separately, removing the liner layer on the silicone adhesive (2477 P, 3 M Inc.) prepared the device. Alignment marks on the device shunt facilitated its precise placement over the shunt, and gentle pressure for 10 s activated the adhesive and laminated it onto the skin. A contact protocol (Supplementary Figure 18) allowed for the identification of poor skin-contact and aberrant sensor behavior. Poor contact resulted in reduced thermal transport away from the actuator and a comparatively large increase in temperature for ~60 s after initiating the thermal actuator: at *t* = 60 s, *T*_US_ and *T*_DS_ were <1 K in cases with good contact, and >2 K in cases with poor contact. A temperature rise across all four NTCs of 1.5 K served as an indication of good contact. As with benchtop studies, the devices thermally equilibrated with the skin temperature for a 120 s period before measurements. Operation of the actuator initiated the period of data collection. In-hospital patients were at reclining angles between 180° (supine) and 90° (upright) during the measurements. Out-patients were at a range of positions and activity levels, as the protocol demanded. In all cases, the device was also mounted on a suitable “off-shunt” location (typically the base of the pectoral muscle) devoid of near-surface anisotropy following the protocol outlined above, as a control. All patients provided written, informed consent to take part in the study.

### Reporting summary

Further information on research design is available in the Nature Research Reporting Summary linked to this article.

## Supplementary information

Supplementary Information

Reporting Summary

## Data Availability

All data needed to evaluate the conclusions are present in the paper and/or in the Supplementary Materials. Additional data information and materials may be requested from one of the corresponding authors.

## Code availability

All custom code was written in MATLAB and is available upon reasonable request from one of the corresponding authors.
