# Supplementary material for: Continuous, noninvasive wireless monitoring of flow of cerebrospinal fluid through shunts in patients with hydrocephalus
Source: NPJ Digit Med. 2020 Mar 6;3:29. doi: 10.1038/s41746-020-0239-1 (PMC7060317; doi:10.1038/s41746-020-0239-1)
Supplement: Supplementary file 1 — Supplementary Information [file 41746_2020_239_MOESM1_ESM.pdf]

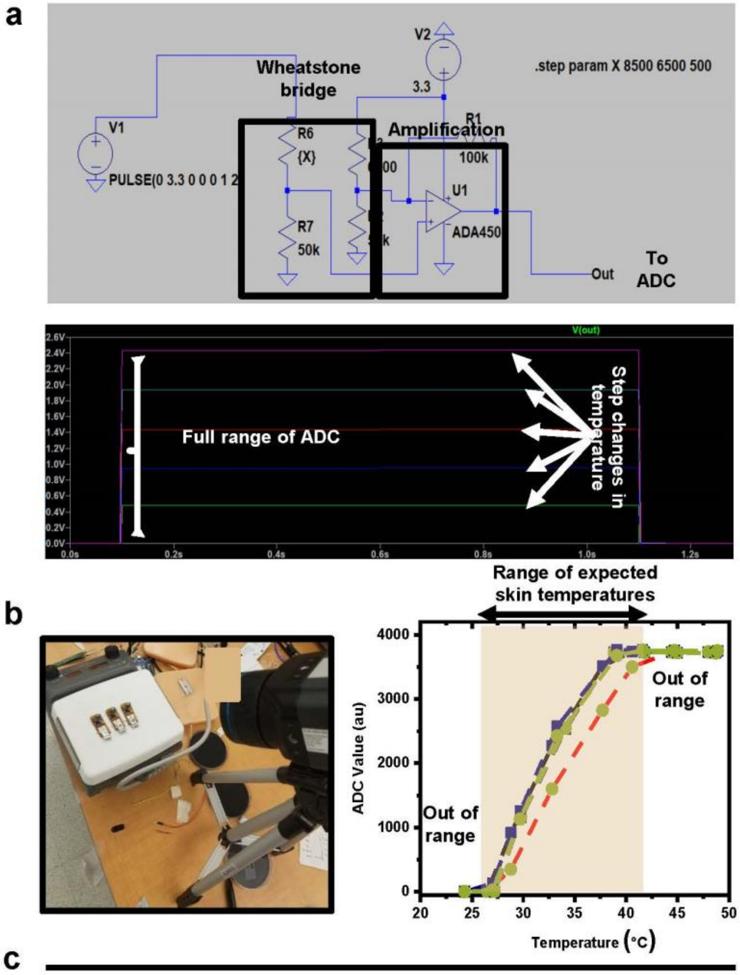

c

Device	Sensor	Slope (˚C/adc unit)	Intercept (˚C)	R^2(Linear best fit in measurement range)
1	1	0.0034	25.468	0.996
1	2	0.0036	27.38	0.9928
2	1	0.0035	25.616	0.9952
2	2	0.0035	25.533	0.9999
3	1	0.0033	25.97	0.9983
3	1	0.0033	26.31	0.9988

Supplementary Figure 1. Precision in temperature sensing in analog front-end circuit. A. LTSpice circuit schematic (top) and simulation (bottom) illustrating change in measured voltage induced by physiologically relevant temperature changes. **B.** Optical image of benchtop calibration system with hot plate and IR camera (left) with calibration curves for 6 temperatures sensors across three devices. **C.** Table summarizing calibration, suggesting measurement precision in temperature of 3.5 mK and measurement threshold of >25˚C.

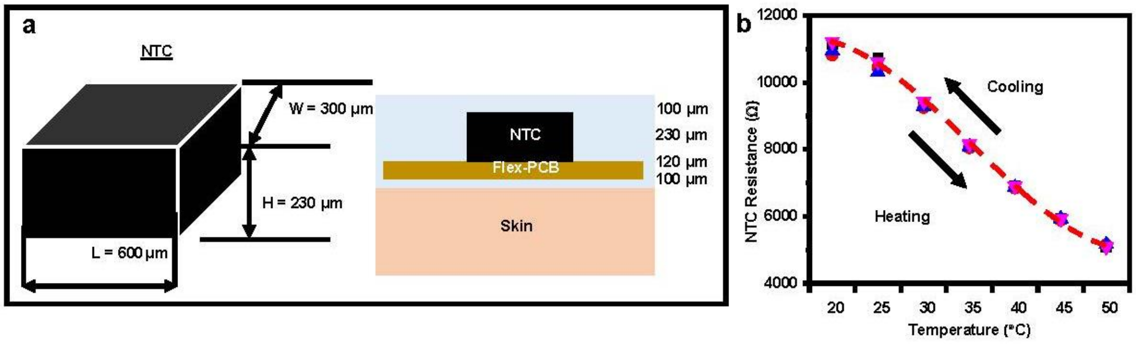

Supplementary Figure 2. Temperature-resistance characteristics of commercially available negative temperature coefficient (NTC) sensing elements. A. Schematic illustration of individual NTC element (left) and NTC incorporated onto flexible circuit board and encapsulated in soft, silicone material (right). **B.** Calibration curves on 3 sensors across successive heating and cooling cycles on hot plate, with temperature measured by IR camera and resistance measured by wired data acquisition system.

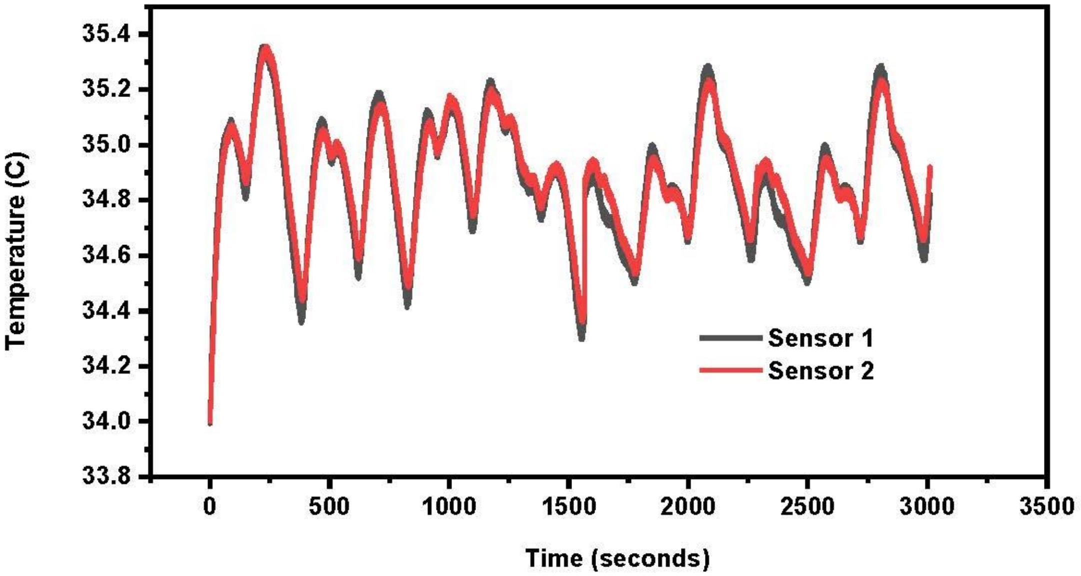

Supplementary Figure 3. Temperature measured by NTC element over 3000s on hot plate set at 40˚C illustrating minimal drift in measurement.

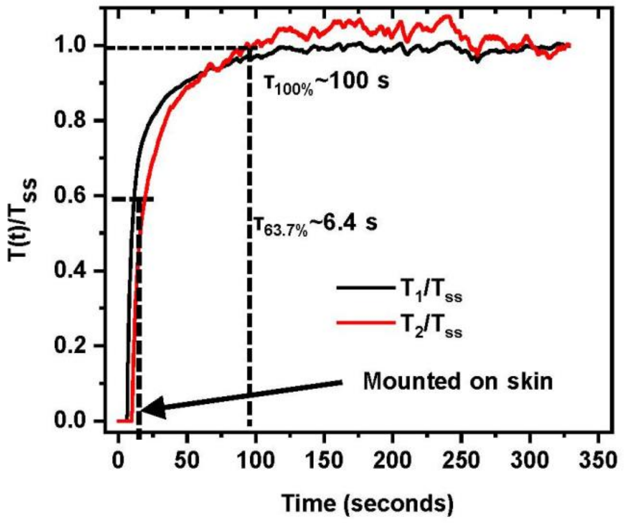

Supplementary Figure 4. Temperature response of hot-plate integrated into flexible circuit board and silicone assembly in response to a step-change in temperature induced by contact with drop of liquid water at 35˚C after equilibrating at 22˚C.

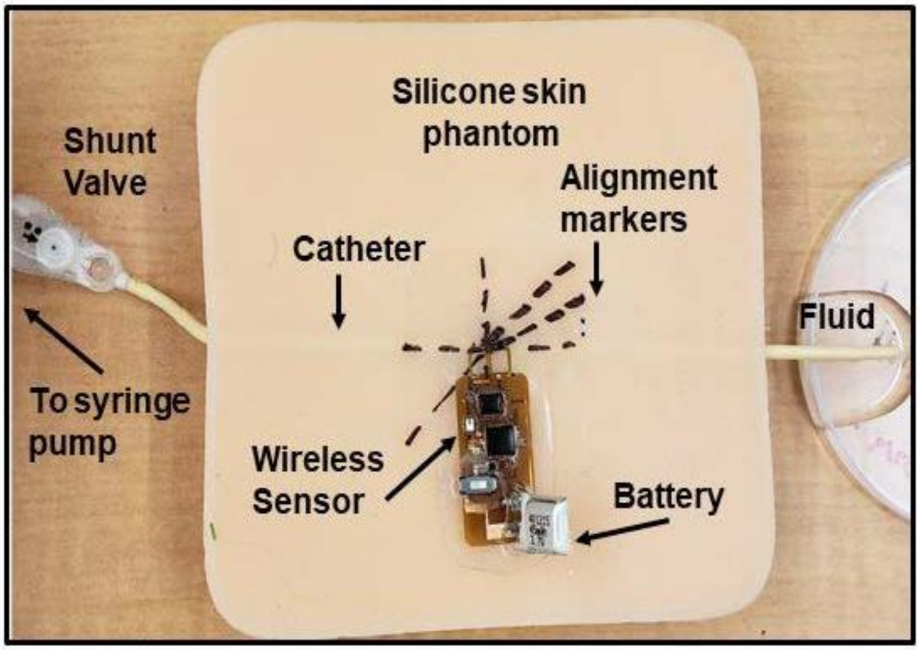

Supplementary Figure 5. Optical image of benchtop silicone shunt phantom assembly.

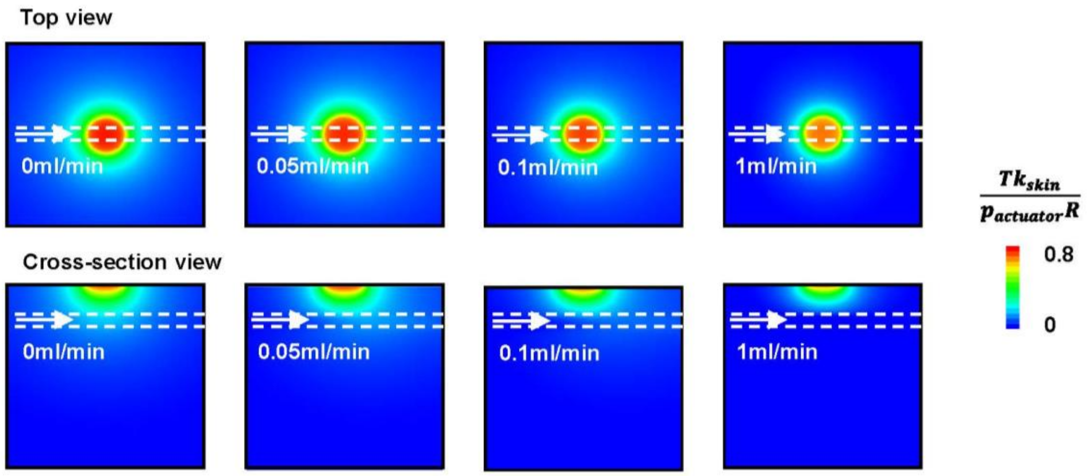

Supplementary Figure 6. 3D Finite Element Models of heat-flow through near-surface layers of skin from actuator, in the presence of underlying flow.

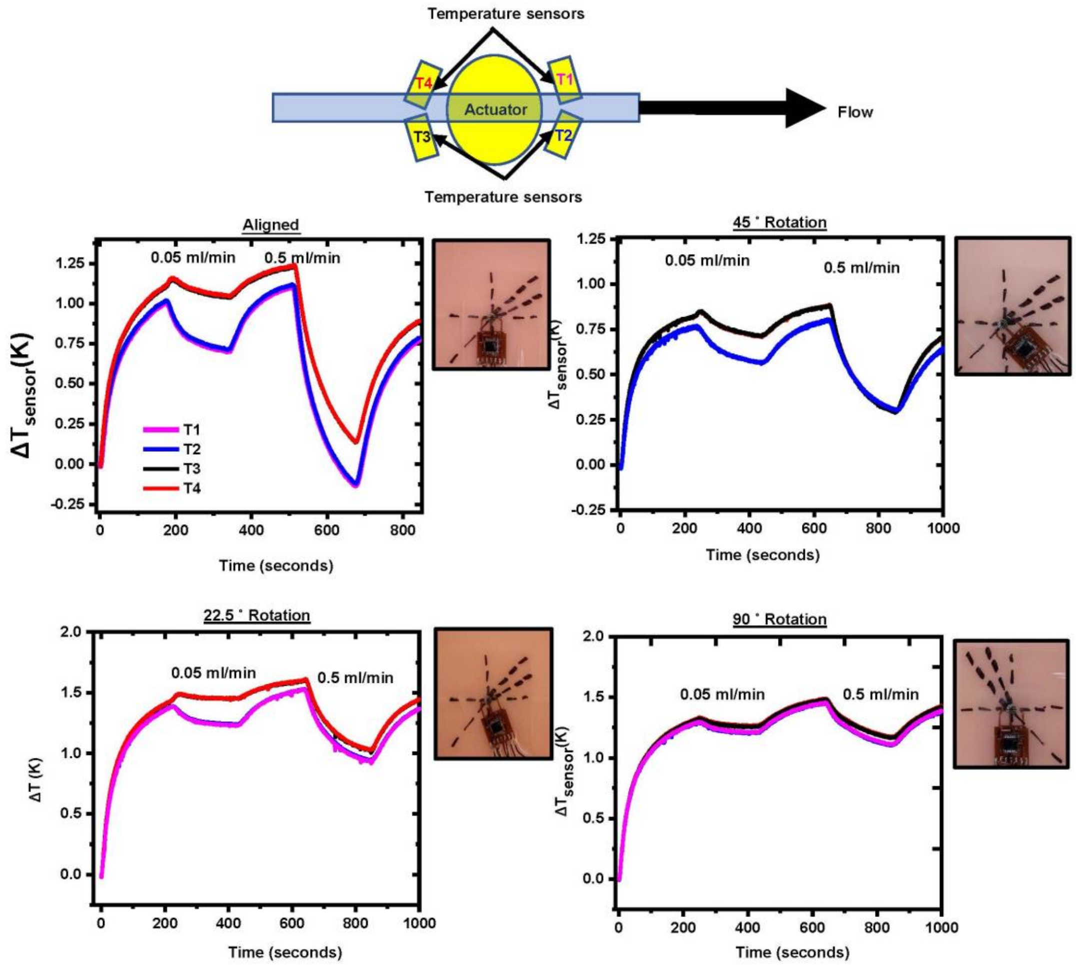

Supplementary Figure 7. Rotational tolerance measured at different angles associated with misplacement of sensor measured on benchtop shunt assembly for two flow rates (0.05 ml/min, 0.5 ml/min) relevant to physiological CSF Flow.

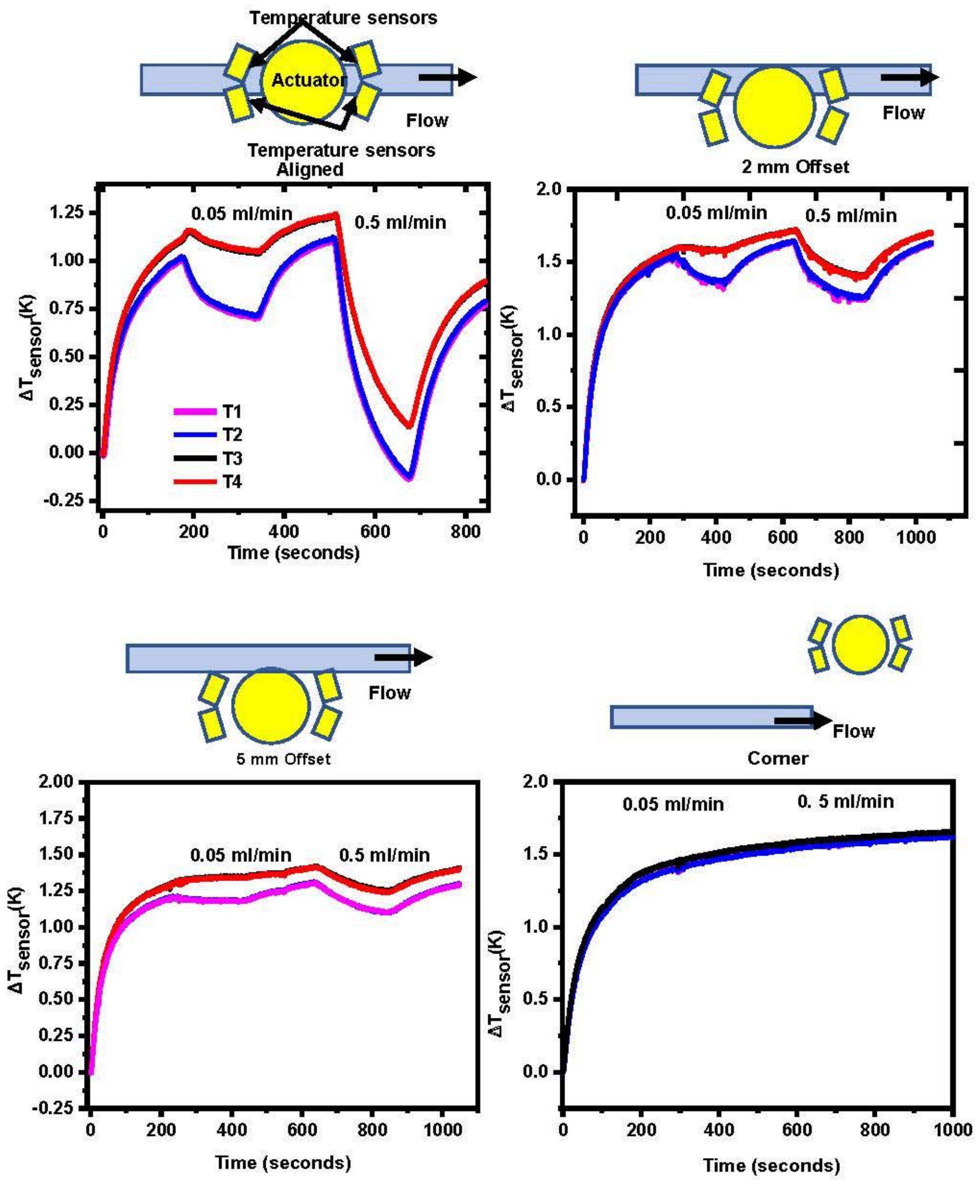

Supplementary Figure 9. Translational tolerance measured at different distances associated with misplacement of sensor measured on benchtop shunt assembly for two flow rates (0.05 ml/min, 0.5 ml/min) relevant to physiological CSF Flow.

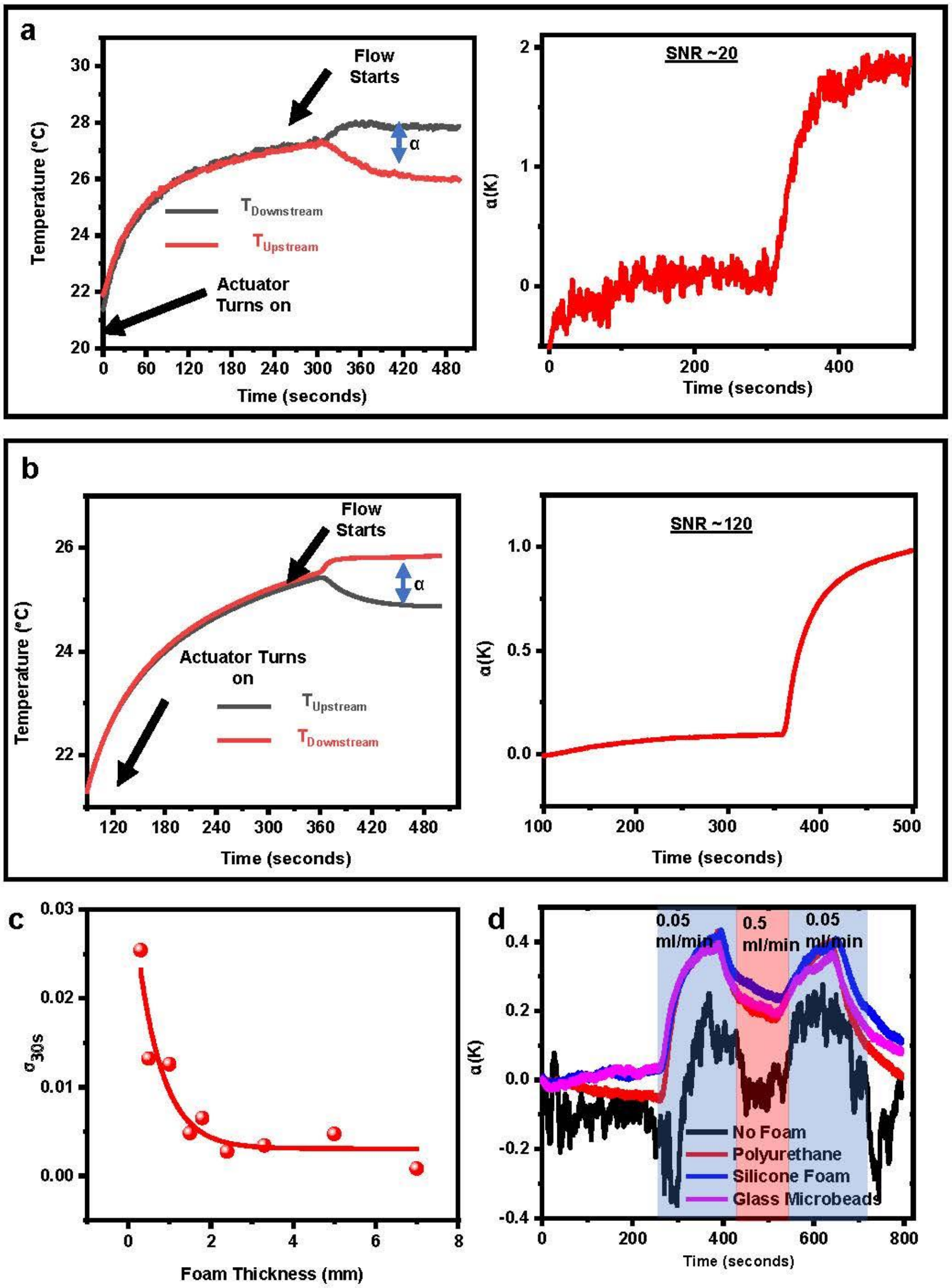

Supplementary Figure 10. Improvement of signal to noise ratio (SNR) with incorporation of thermally insulating foam layer. A. Raw temperature measurements on benchtop assembly before and during flow, without insulating foam (left) with computed values of α (right). **B.** Same as **A.**, but with thermally insulating foam. **C.** Noise in system, as measured by standard deviation in computed values of α over a 30s sampling window, as a function of foam thickness. **D.** α measured for different foam formulations, including polyurethane (red), silicone (blue) and a silicone-silica microsphere mixture (pink) and a no-foam case (black) at two flow rates, 0.05 ml/min and 0.5 ml/min.

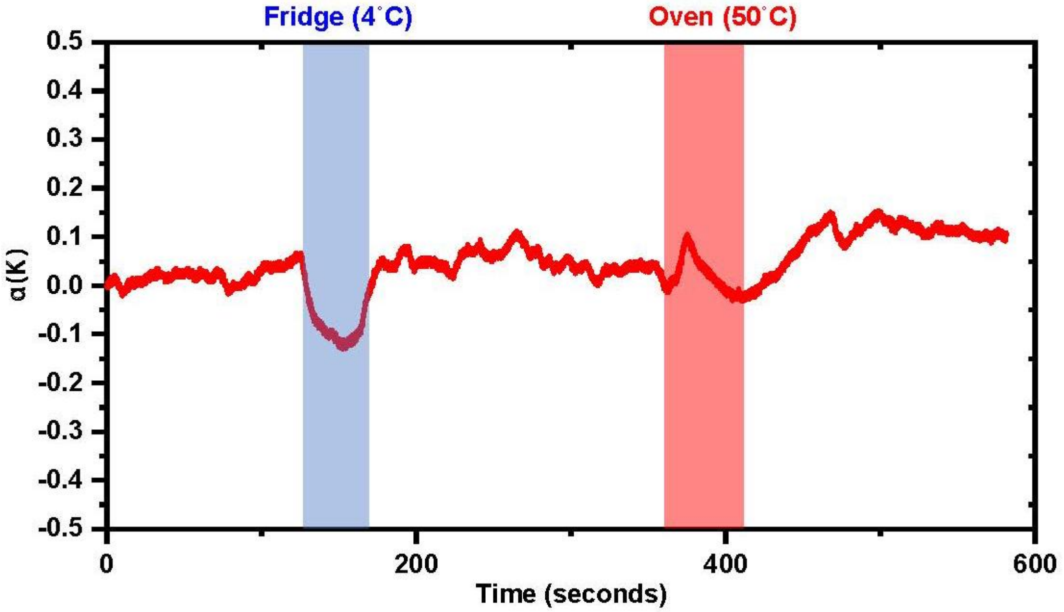

Supplementary Figure 11. Values of α measured continuously on a subject's volar forearm as subject moves from room temperature to fridge environment at 4˚C and an oven at 50˚C.

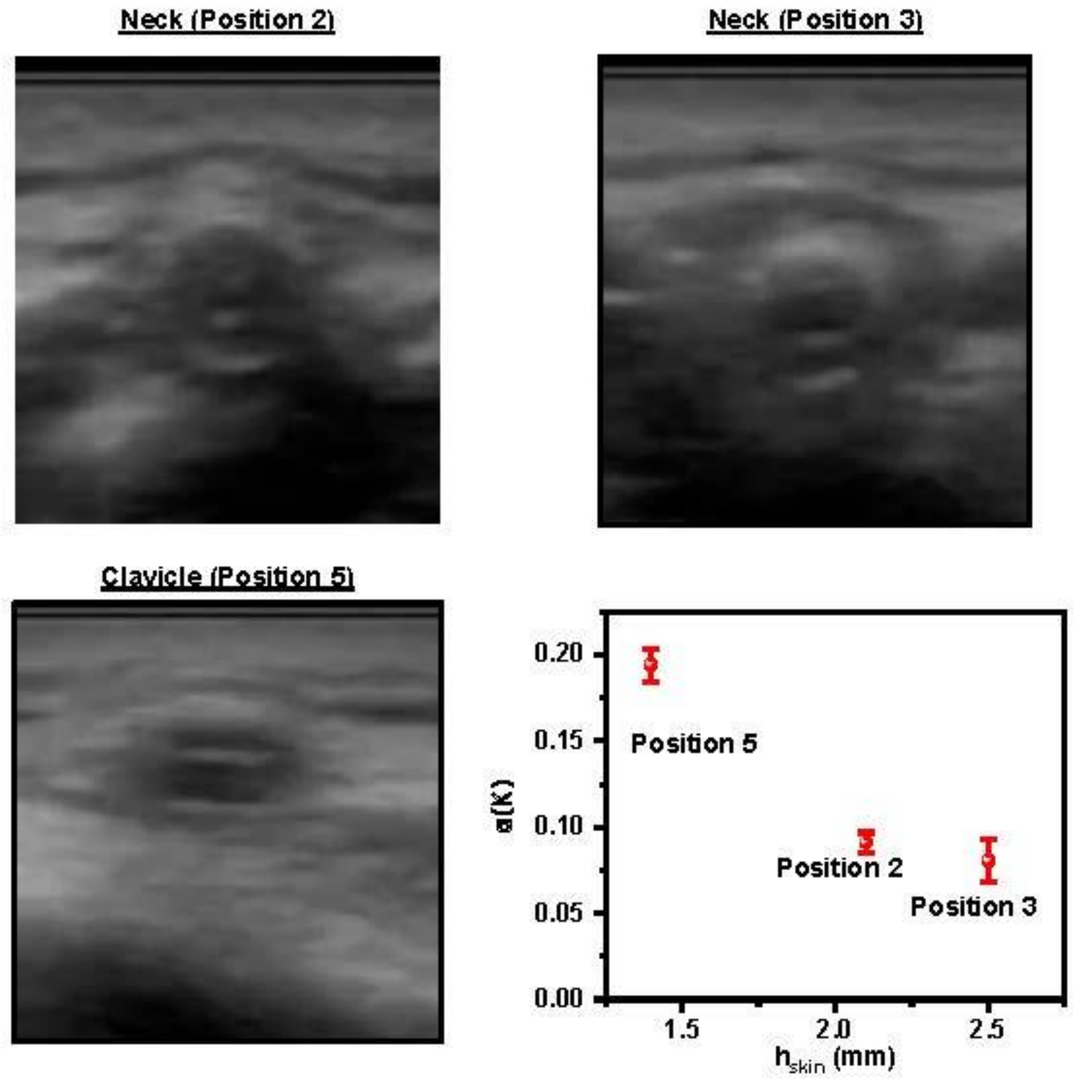

Supplementary Figure 12. Ultrasound images measured at three different positions on neck, with different skin thicknesses and corresponding values of *α* measured successively and averaged over a 100s window. Error bars correspond to average values over 100s.

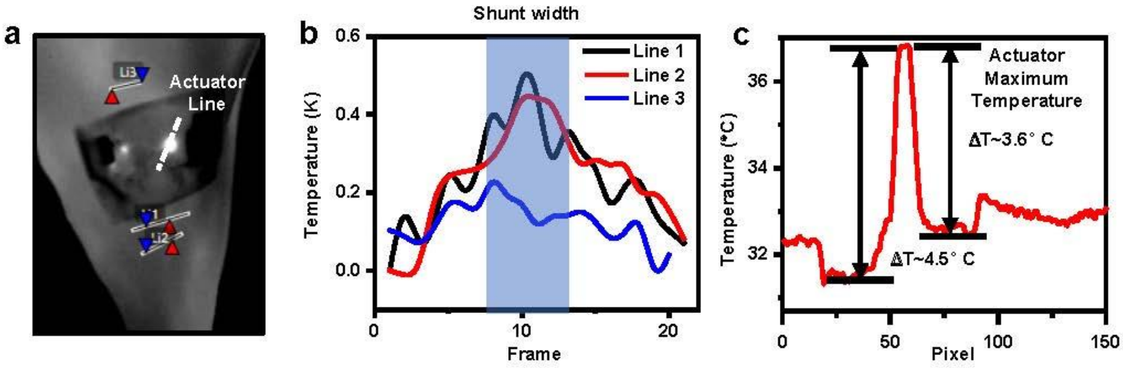

Supplementary Figure 13. IR Imaging on patient. A. IR thermograph of patient with operational device mounted over shunt. **B.** Linear temperature profile at two downstream locations (Line 1, Line 2) and one upstream location (Line 3) perpendicular to direction of CSF flow through shunt, and directly over shunt, **C.** Linear temperature profile across actuator line, illustrating maximum temperature rise of 4.7˚C across actuator.

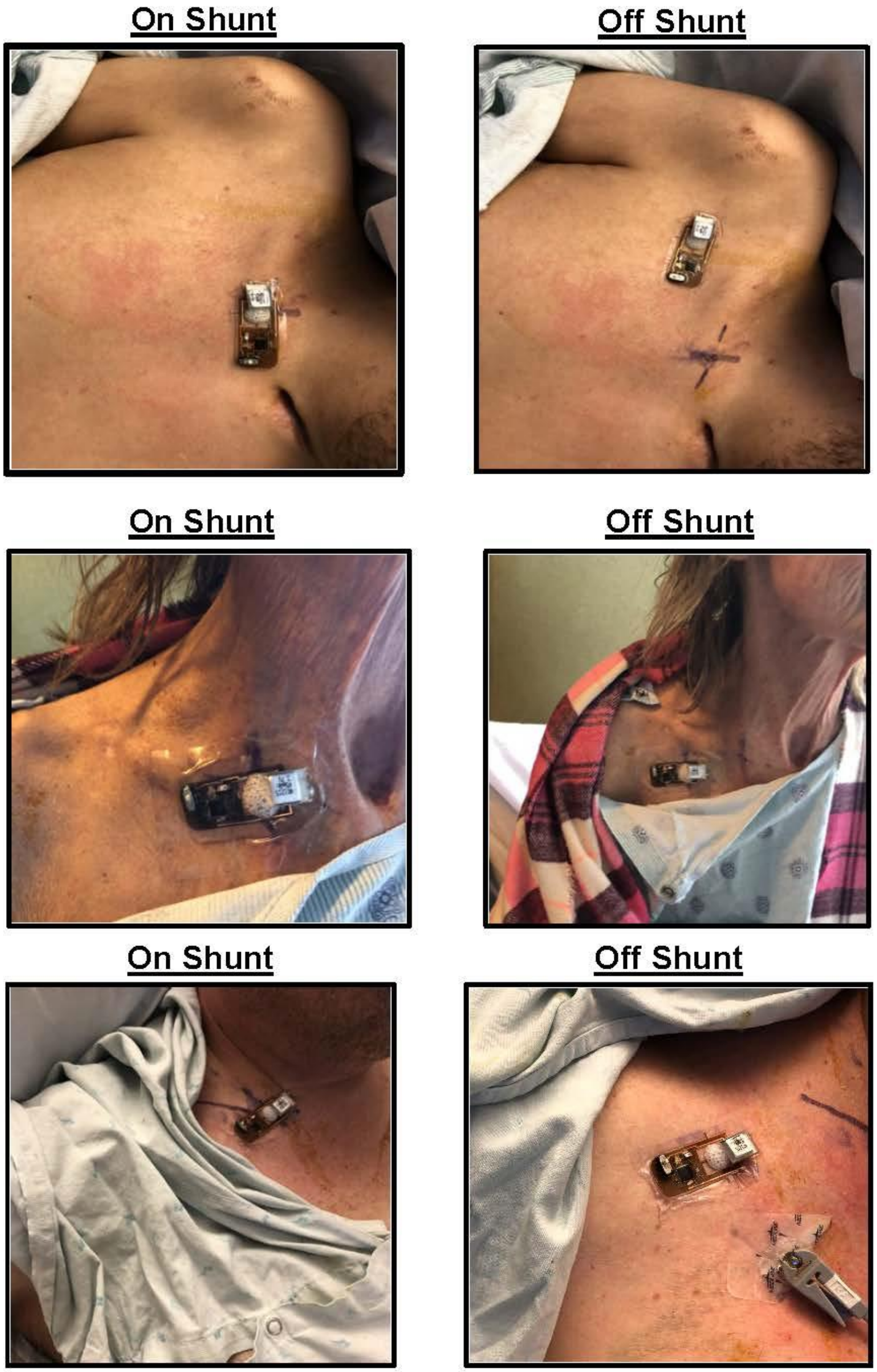

Supplementary Figure 14. Clinical images on patients. Participants provided written informed consent to have their photos/images included as a part of this publication.

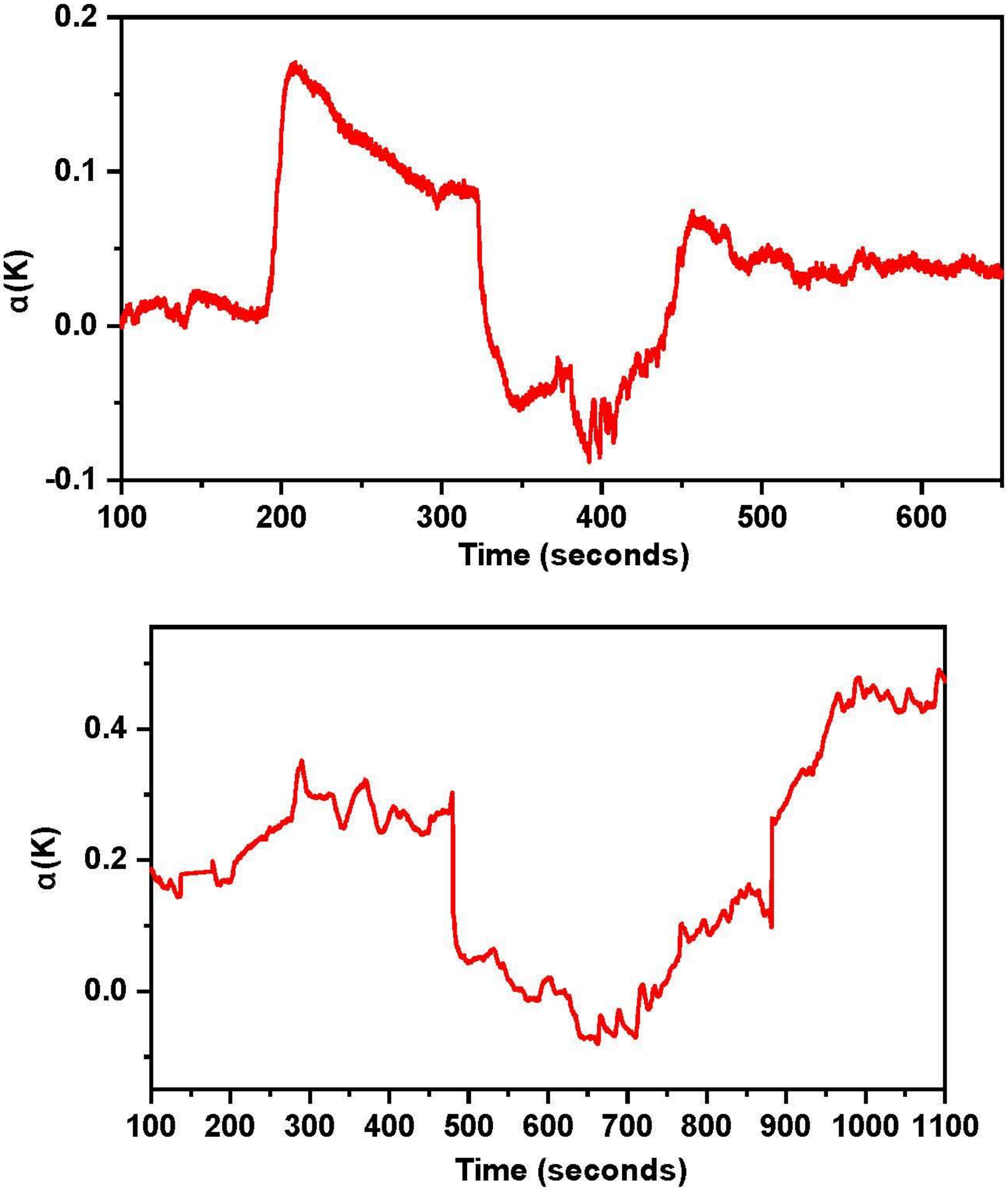

Supplementary Figure 15. Raw data for *α(t)* on 2 patients corresponding to calculations in Fig. 5B.

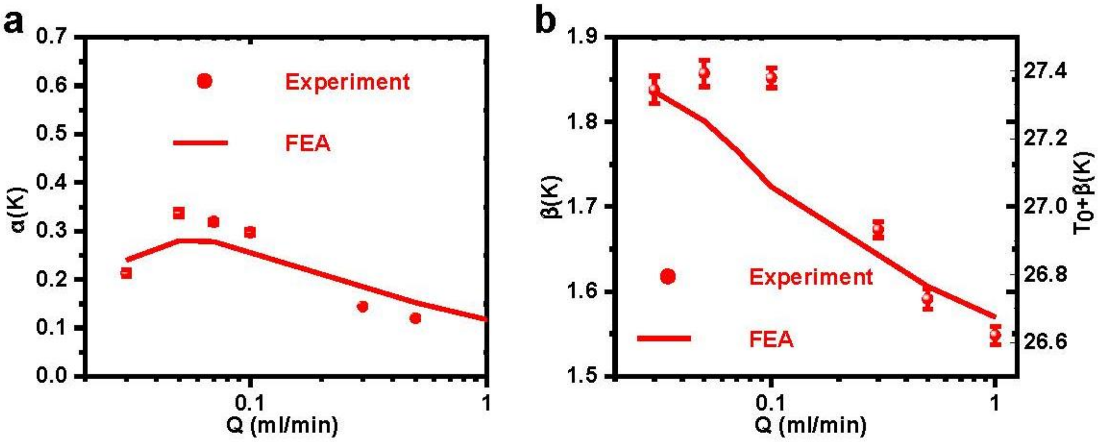

Supplementary Figure 16. Experimental (line) and FEA-computed (line) data for α and β on silicone skin phantom ($k_{silicone}$=0.21 W/m-K) for h_{skin}=1.7 mm.

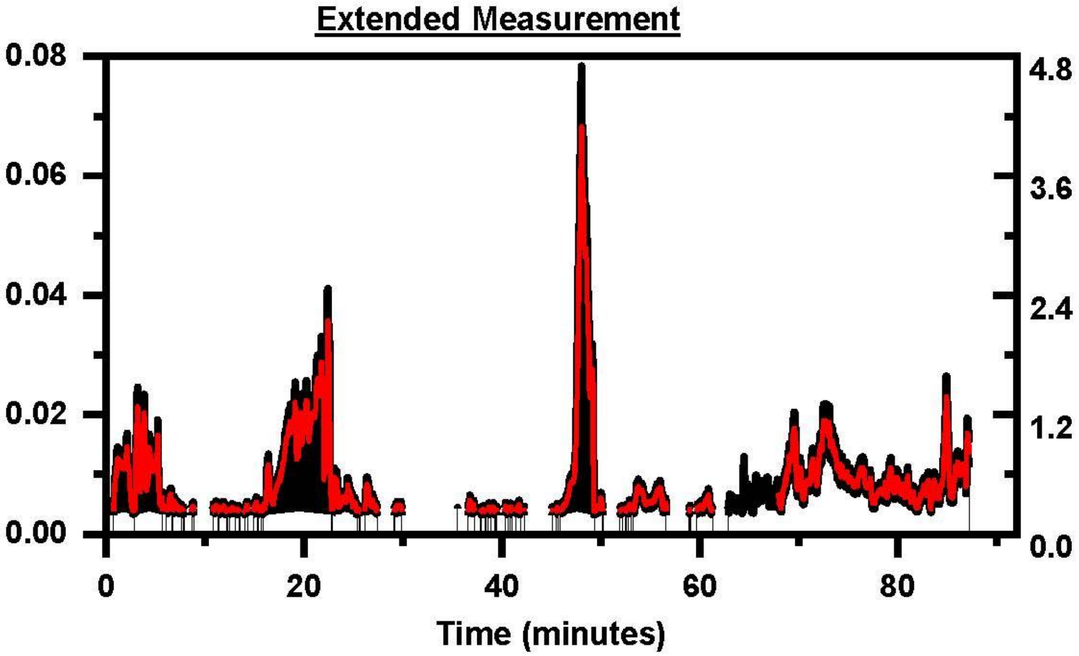

Supplementary Figure 17. Extended measurement of Q computed from α conversion equations. Values of Q corresponding to α<0.01K are omitted due to breakdown of fit.

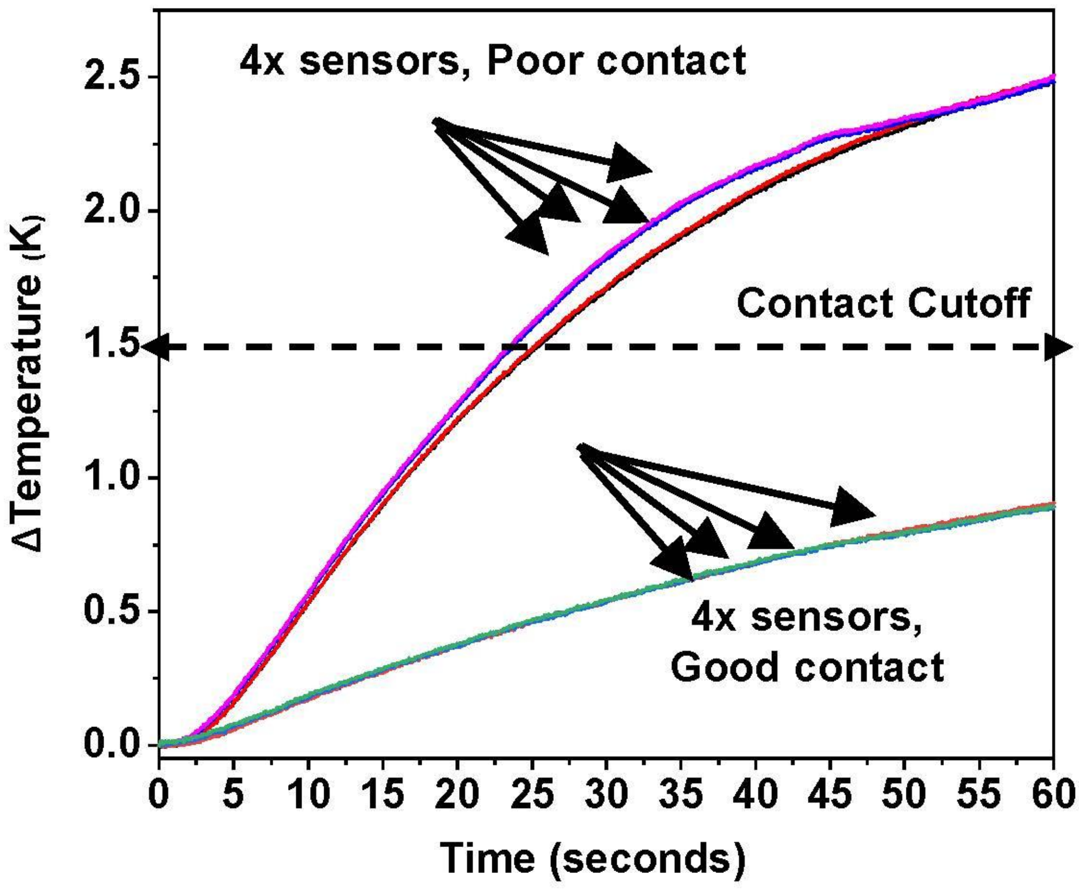

Supplementary Figure 18. T_{DS} **and** T_{US} **for 60s after actuation for cases with good skin contact and poor skin contact on neck of healthy outpatient, illustrating enhanced local temperature rise owing to poor thermal transport at skin-device interface, and cutoff point (1.5K at 60s) for poor contact.**
